# Initiation of HIV Reverse Transcription

**DOI:** 10.3390/v2010213

**Published:** 2010-01-18

**Authors:** Catherine Isel, Chantal Ehresmann, Roland Marquet

**Affiliations:** Architecture et Réactivité de l’ARN, Université de Strasbourg, CNRS, IBMC, 15 Rue René Descartes, 67084 Strasbourg cedex, France; E-Mail: Chantal.Ehresmann@free.fr (C.E.)

**Keywords:** retrovirus, reverse transcriptase, tRNA

## Abstract

Reverse transcription of retroviral genomes into double stranded DNA is a key event for viral replication. The very first stage of HIV reverse transcription, the initiation step, involves viral and cellular partners that are selectively packaged into the viral particle, leading to an RNA/protein complex with very specific structural and functional features, some of which being, in the case of HIV-1, linked to particular isolates. Recent understanding of the tight spatio-temporal regulation of reverse transcription and its importance for viral infectivity further points toward reverse transcription and potentially its initiation step as an important drug target.

## 1. Introduction

Reverse transcription is a central event in the retroviral life cycle, allowing conversion of the single-stranded genomic RNA into a double-stranded DNA with duplicated long terminal repeats [1]. This is achieved by the viral reverse transcriptase (RT) that possesses an RNA- and DNA- dependent DNA polymerase activity as well as an endonuclease activity (RNase H) [2]. In both HIV-1 and HIV-2, DNA synthesis is initiated by the cellular tRNA_3_^Lys^ selectively packaged into the virion [3]. The tRNA_3_^Lys^ packaging process has been recently unravelled [4]: it involves a number of interactions between a Gag/Gag-Pol/vRNA entity and the mitochondrial lysyl-tRNA synthetase (LysRS)/tRNA_3_^Lys^ complex. In order for reverse transcription to start, the 3′-terminal 18 nucleotides of the primer tRNA are annealed to the complementary viral sequence called the Primer Binding Site (PBS) present in the 5′-untranslated region (UTR) of the genomic RNA. The precise timing, *i.e*., during the budding process or during maturation of the viral particles, and the number of steps of this process still remain to be determined. Formation of the vRNA/tRNA_3_^Lys^ complex cannot occur spontaneously at physiological temperature since both RNA molecules involved are highly structured. Hence, a viral chaperone protein, namely the nucleocapsid protein (NCp) is involved in formation of the HIV-1 initiation complex of reverse transcription. Recent *in vitro* data showed that the Viral Infectivity Factor (Vif) can partially replace NCp in this function, while the restriction factors APOBEC3G (A3G) and A3F have been proposed to inhibit this process. *In vitro* and *in vivo* structural probing, mutagenesis, as well as replication studies with mutated HIV-1 genomes “forced” to use non-homologous primers indicated that the vRNA/tRNA_3_^Lys^ interactions are not restricted to the PBS and the 18 3′-terminal nucleotides of tRNA_3_^Lys^. However, different groups proposed different interactions, and a consensus has yet to be found. Several studies pointed at the structural versatility of the vRNA/tRNA_3_^Lys^ initiation complex, which might not be conserved among different HIV-1 isolates, and which could adopt different two- and three-dimensional structures when using wild type or mutant vRNA templates and tRNA_3_^Lys^ or other primers. In addition, detailed kinetic studies revealed that the initiation process is clearly distinct, at the enzymatic level, from the subsequent elongation step. Our current understanding of the initiation complex of HIV-1 reverse transcription has driven some interest in using this step as a target for new and specific drugs. This is all the more relevant that recent work has highlighted the importance of a tight spatio-temporal regulation, by viral co-factors such as NC, of the reverse transcription process, in order for the virus to maintain its infectivity.

## 2. Selective packaging of the primer tRNA

Different retroviruses use different tRNA primers, all of cellular origin (for review, see [3]). Some lentiviruses use tRNA_1,2_^Lys^ as a pimer for reverse transcription, other use tRNA_3_^Lys^. For HIV-1, tRNA_3_^Lys^ is the primer and is “selectively” packaged into the viral particles. The “selective” packaging refers to the enrichment of the primer tRNA species packaged into the virion by comparison with the tRNA population present in the cytoplasm of the infected cell [5]. For example, in particles produced from COS7 cells transfected with a HIV-1 proviral DNA, the three major isoacceptors of tRNA^Lys^ (tRNA_1,2_^Lys^ and tRNA_3_^Lys^) were preferentially packaged, with the relative concentration of tRNA^Lys^ increasing from 5–6 % in the cell to 50–60 % in the virion, with respect to low molecular weight RNA species [6–8]. The number of tRNA^Lys^ packaged was estimated at 20–25 molecules per virion, with the same tRNA_3_^Lys^/tRNA_1,2_^Lys^ ratio as in the cell [9].

### 2.1. Structure of tRNA_3_^Lys^

**Figure 1.**
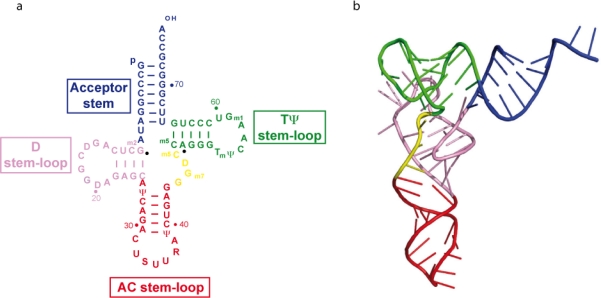
Secondary **(a)** and tertiary **(b)** structures of tRNA_3_^Lys^.

tRNA_3_^Lys^ folds into a classical two-dimensional cloverleaf structure, with acceptor, D, anticodon and TΨC arms (Figure 1a). The three-dimensional structure of tRNA_3_^Lys^ has been solved to 3.3 Å resolution [10]. As expected, tRNA_3_^Lys^ adopts the canonical L-shaped structure that was first highlighted in yeast tRNA^Phe^ (Figure 1b). More importantly, the crystal structure reveals that the anticodon loop of human tRNA_3_^Lys^ is also perfectly canonical. This contradicts early findings from two different groups, both of which have been studying, by NMR, the solution structure of either an undermodified pentanucleotide mimicking the anticodon loop [11] or a much larger oligonucleotide, lacking any modifications at positions U34 and A37 [12]. The recently published NMR structure of an entire, unmodified, tRNA_3_^Lys^ [13] is in complete agreement with the standard three-dimensional structure of a tRNA revealed by X-ray crystallography.

### 2.2. The role of Gag-Pol

**Figure 2.**
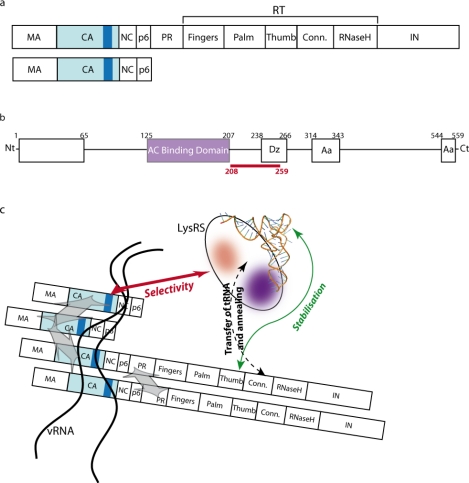
Partners and model for the tRNA_3_^Lys^ packaging complex **(a)** Organisation of the Gag-Pol and Gag precursors. MA: matrix ; CA: capsid ; NC: nucleocapsid ; p6: HIV-1 p6 protein ; PR: protease; RT: reverse transcriptase, with its Fingers, Palm, Thumb, Connection (Conn.) and RNase H domains; IN: integrase. The dark blue rectangle in the capsid domain of the precursors corresponds to the C-terminal helix 4 that was shown to interact with lysyl-tRNA synthetase (LysRS) **(b)** Organisation of LysRS: the anticodon (AC) binding domain is located between positions 125 and 207 and the LysRS dimerisation (Dz) domain between positions 238 and 266. The two boxes toward the C-terminus of the protein, between positions 314 and 343 and 544 and 559 are important for amino acid recognition. The area highlighted in red (208–259), overlapping the dimerisation domain, is involved in binding to the capsid. **(c)** Model for the packaging complex. The purple patch corresponds to the anticodon binding domain and the red patch to amino acids 208–259 that interact with the CA domain of Gag.

Selection of tRNA^Lys^ does not depend on viral RNA packaging or maturation of protein precursors [9], but requires the unprocessed Gag and Gag-Pol precursors (Figure 2a) [8,14,15]. Since RT interacts with primer tRNA_3_^Lys^ during reverse transcription, the RT domain of Gag-Pol had been proposed as a likely candidate for interacting with tRNA^Lys^ during selective packaging. Accordingly, the thumb domain of RT, shown to interact with tRNA_3_^Lys^ *in vitro* [16–18], was also shown to be important for *in vivo* tRNA packaging [19]. However, the putative implication of the tRNA^Lys^ anticodon/RT thumb interaction [16, 18] has been questioned, since mutations in the RT thumb domain at positions found to inhibit the interaction of mature RT with the tRNA_3_^Lys^ anticodon *in vitro* [16], did not affect tRNA_3_^Lys^ packaging *in vivo* [19]. A recent model suggests that the interaction between the thumb domain of RT within the Gag-pol precursor and tRNA_3_^Lys^ only plays a stabilizing role [20].

### 2.3. The role of LysRS

In mammalian cells, LysRS (Figure 2b), which aminoacylates tRNA^Lys^, is involved in a multi-aminoacyl tRNA-synthetase complex comprising 11 enzymes, amongst which only LysRS is found in HIV-1 viral particles [21–23]. Notably, about 25 molecules of LysRS are found per virion, a number close to that of tRNA^Lys^ molecules, suggesting the existence of an equimolar complex between these two partners. However, and importantly, the incorporation of LysRS into the virion does not require tRNA^Lys^ packaging [22]. On the other hand, packaging of tRNA^Lys^ appears to be controlled by LysRS, since the amount of primer tRNA that is encapsidated is affected by variations in the number of packaged LysRS molecules [23–25]. In addition, RNA interference experiments designed to decrease cellular expression of LysRS not only reduced tRNA^Lys^ packaging, but also viral infectivity [25]. Precise mapping revealed that the N-terminal tRNA binding domain of LysRS [26–28], as well as the tRNA_3_^Lys^ anticodon known to be the major determinant for LysRS recognition [29], is required for tRNA packaging.

Newly synthesized cytoplasmic LysRS was long believed to be the form that is encapsidated into the HIV-1 viral particles. Human cytoplasmic and mitochondrial LysRSs result from the same gene and are produced by alternative splicing, with the very N-terminus of the two forms being different. Production of antibodies able to discriminate between the two proteins pointed to the mitochondrial enzyme, in either its pre-mitochondrial or mature form lacking the signal peptide which is cleaved during import, as the packaged enzyme [30]. Selective packaging of the mitochondrial form of LysRS into the viral particles could be explained by the fact that the cytoplasmic LysRS is involved in the multi-aminoacyl tRNA-synthetase complex, which would impede its interaction with HIV or other targeting proteins.

### 2.4. The packaging complex

Gag alone was proven to be sufficient for LysRS packaging [22] and Kleiman and co-workers mapped the Gag/LysRS interactions *in vitro* and *in vivo*. Their results revealed that the two partners interact through the dimerisation domains of both LysRS and the CA domain of the Gag precursor [31]. Fine mapping indicated that Helix 7 of the LysRS dimerisation domain interacts with Helix 4 of the C-terminal dimerisation domain of the CA [32,33]. This interaction is thought to be responsible for the selectivity of the packaging process. A weaker interaction between LysRS and the thumb-RNase H domain of RT, only observed when both peptides were expressed in the cell, was also reported [20]. Based on the data described so far, a model for the packaging complex has been proposed [4,5] (Figure 2c). In this model, two sub-complexes are involved: 1) the Gag/Gag-Pol/vRNA complex is maintained together *via* well documented Gag/Gag interactions (occurring between the CA, NC and p6 domains [34]), Gag-Pol/Gag-Pol interactions (occurring via the PR dimerisation domain [35]) and Gag/vRNA interactions [36–40]; 2) the tRNA_3_^Lys^/LysRS complex is maintained together *via* an interaction between the anticodon of tRNA_3_^Lys^ and the N-terminal anticodon binding domain of the synthetase [4]. As mentioned before, the two complexes interact via a Gag/LysRS interaction that is responsible for the selective incorporation of LysRS and hence tRNA_3_^Lys^ into the viral particles, whereas the thumb-RNase H domain of RT within Gag-Pol only stabilizes the interaction [20]. Finally, it was shown that although the RT connection domain is dispensable for tRNA packaging, it is required for its annealing to the vRNA [41].

### 2.5. tRNA_3_^Lys^ amino-acylation

While previous studies indicated that, on the contrary to cellular tRNA^Lys^, tRNA^Lys^ purified from virions is uncharged [9], it is not clear whether charged or uncharged tRNA^Lys^ is initially packaged. It was shown that the mature HIV-1 RT was unable to extend acylated tRNA_3_^Lys^ and did not increase deacylation [42]. Thus, a possible role of tRNA_3_^Lys^ acylation could be to prevent premature initiation of reverse transcription [42,43]. The fact that only deacylated tRNA_3_^Lys^ was found within viral particles does not contradict this assertion, since spontaneaous deacylation of tRNA_3_^Lys^ could easily occur during the time lapse necessary for virion collection and purification.

## 3. Secondary structure of the PBS domain

Detailed *in vitro* and *in situ* structural studies were conducted on HIV-1 MAL, a recombinant virus with a PBS domain of subtype A origin [43,44], and subtype B isolates Lai, HxB2 and NL4-3 [44–47]. Although the different isolates display strong sequence homologies, the MAL isolate differs from the subtype B isolates by a 23-nucleotide insertion downstream of the PBS (nucleotides 211 to 233, underlined in figure 4b) resulting from a partial duplication of the PBS sequence and several point mutations (Figure 4b, d). Sequence alignments reveal that 14% of all HIV-1 isolates possess this insertion [48]. However, as this insertion is absent from subtype B isolates, which are over-represented in the databank, but present in the subtypes and circulating recombinant forms most actively involved in spreading of HIV-1, the biological importance of isolates presenting a MAL-like insertion is greater than suggested by the databank [48]. These sequence differences account for the different secondary structure models proposed by different groups, with a conserved A-stretch located 10 nucleotides upstream of the PBS either in an apical loop in the case of MAL (Figure 4b) [44,49] or in an internal loop for the Lai, NL4-3 or HxB2 isolates [39,44–47,50–52] (Figure 4d). Notably, the secondary structures shown in Figure 4b and 4d, first deduced from classical *in vitro* probing studies, are supported by recent experiments conducted on viral RNA directly modified within infected cells [45–48].

## 4. The Primer/Template Complex

### 4.1. From initiation to elongation

**Figure 3.**
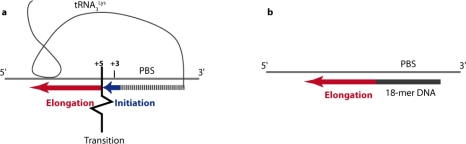
Schematic representation of HIV-1 specific initiation **(a)** *versus* unspecific elongation **(b)** of reverse transcription. The vRNA template is represented by a thin grey line. The natural tRNA_3_^Lys^ primer or an 18 mer DNA primer are in black and the newly synthesized DNA is represented by thick blue and red lines representative of the initiation and elongation steps of reverse transcription, respectively. In the presence of the natural tRNA primer, transition between initiation and elongation occurs after the addition of the first 6 nucleotides to the 3′ end of the primer. In the case of the HIV-1 MAL isolate, transition is facilitated by the anticodon/A-rich loop interaction upstream of the PBS, represented by the close contact between tRNA_3_^Lys^ and the vRNA.

When reverse transcription was tested *in vitro* using purified natural tRNA_3_^Lys^, a synthetic vRNA fragment and purified recombinant RT, it appeared that (-) ssDNA synthesis proceeds in a two step manner [53–56] (Figure 3): 1) an initiation phase, during which RT dissociates rapidly from the primer/template duplex, corresponds to the slow and distributive addition of the first 6 nucleotides. Initiation was found to be specific, since non-homologous RTs, like AMV and MLV RTs, did not extend the natural tRNA_3_^Lys^ primer efficiently; 2) an unspecific elongation phase, where dissociation of RT is slow, leading to processive DNA synthesis. During elongation, there is no specific recognition between HIV-1 RT and the primer/template duplex and any RT performs in the same manner. It was shown that the modified nucleotides of the natural tRNA were required for the specificity and efficiency of the initiation step [53–55,57] and that a DNA oligonucleotide strictly complementary to the PBS was representative of the elongation step (Figure 3). Such features were also demonstrated for HIV-2 [58] and FIV [56], and could be a general characteristic of all retroviruses, since there is also evidence from avian and murine retroviruses [59].

### 4.2. Extended HIV-1 RNA/tRNA_3_^Lys^ interactions and their implication in initiation of reverse transcription *in vitro*

The idea that the PBS is not the sole determinant for primer usage was supported by observations from different laboratories. Indeed, transfection of proviral DNA with PBS sequences mutated to be complementary to the 3′ end of tRNAs other than the natural tRNA_3_^Lys^ yielded HIV-1 viruses with dramatically reduced replication kinetics that eventually reverted back to the wild-type PBS sequence [60–62]. Notably a similar observation was made with avian viruses [63] but not with murine viruses [64,65]. Various intermolecular primer/template interactions in HIV-1 were proposed by different groups to modulate the efficiency of reverse transcription *in vitro*, leading to apparently conflicting results.

#### 4.2.1. Two and three-dimensional structures of a representative subtype A initiation complex

**Figure 4.**
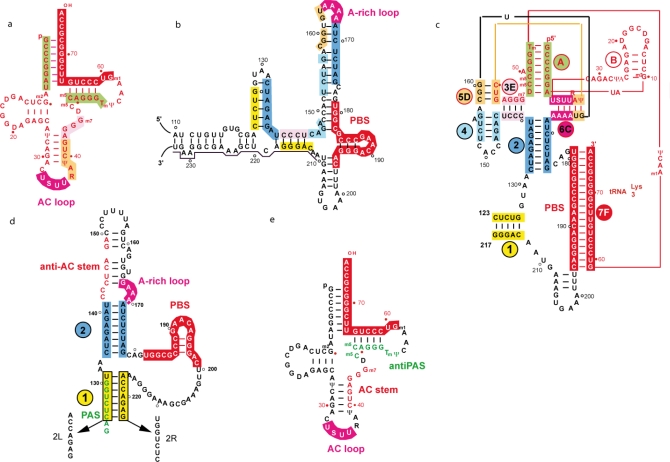
Secondary structure of the RNA partners of the HIV-1 initiation complex of reverse transcription and of the binary primer/template complexes, in the case of the HIV-1 MAL (representative of a subtype A PBS domain) and subtype B isolates. The regions undergoing intra- or intermolecular rearrangements upon formation of the primer/template complex are highlighted in various colours. Boxes or sequences of the same color represent areas that are base-paired in the binary complex. **(a)** The human tRNA_3_^Lys^. **(b)** The PBS sub-domain in the free form of the HIV-1 MAL vRNA. **(c)** The HIV-1 MAL vRNA/tRNA_3_^Lys^ complex. **(d)** The PBS sub-domain in the free form of the HIV-1 NL-4.3 (subtype B) isolate. The PAS and mutations 2L and 2R are indicated. **(e)** Localization of the anti-PAS region of tRNA_3_^Lys^.

The first *in vitro* structural data for a tRNA/vRNA duplex were obtained from enzymatic and chemical probing on a heat-annealed complex between tRNA_3_^Lys^ and a fragment of HIV-1 MAL vRNA containing the complete 5′-UTR [66,67]. They revealed intricate intermolecular interactions between tRNA_3_^Lys^ and viral sequences located upstream of the PBS (Figure 4a-c). An identical conformation of the binary complex was observed when the primer was annealed to the vRNA with the nucleocapsid protein [68]. The stability of the proposed extended interactions was found to depend on the post-transcriptional modifications of tRNA_3_^Lys^ [67,69] and on the complementarity between the anticodon loop of the primer and the conserved A-rich loop of the vRNA located in the apex of the U5 stem [66,70].

The NMR structure of a stem-loop containing the A-rich sequence of the MAL isolate revealed that the structure of the loop resembles the one of a tRNA anticodon loop, with a non-canonical G-A pair closing the loop and a U-turn motif leading to the continuous stacking of 164-AAA-166 onto A167 [71]. This pre-stacked structure renders the loop a good candidate for base-pairing, most likely with the U-rich anticodon loop of tRNA_3_^Lys^, in the same way as any tRNA anticodon would base-pair with messenger RNA. The solution structure of a duplex representative of the A-rich loop/tRNA interaction pointed to the importance of base modifications for stabilization of the vRNA/tRNA complex, in agreement with previously published work [57,67,69]. Indeed, the thio-group of U34 plays a crucial role in the stabilization of the loop-loop interaction that makes helix 6C (Figure 4c), whereas Ψ39 contributes to it [72]. Accordingly, *in vitro* reverse transcription experiments revealed that the modified nucleotides of tRNA_3_^Lys^ and extended primer-template interactions are required for efficient initiation and transition to elongation of reverse transcription respectively [53,57].

A three-dimensional model of the MAL vRNA/ tRNA_3_^Lys^ /RT ternary complex was proposed, based on probing data and footprinting experiments [73]. Strikingly, none of the extended intermolecular interactions (helices 3E, 5D and 6C in Figure 4c) are directly recognized by RT. Their assumed role is to impose a particular primer/template conformation that correctly places the intermolecular helix containing the PBS (helix 7F) and the 3′-OH group of the primer into the RNA-binding cleft of RT. Mutational analysis pointed to the three-nucleotide junction between helices 2 and 7F and the intermolecular RNA/tRNA interactions forming helix 6C as the most critical elements for efficient initiation of reverse transcription (Figure 3c) [44,53]. These results are in keeping with the three-dimensional model and agree with the idea that the intermolecular interactions impose a precise tertiary structure and/or counteract possible inhibitory effects of the vRNA structure that could otherwise lead to steric clashes between RT and RNA.

#### 4.2.2. Secondary structure of tRNA/vRNA complexes representative of subtype B isolates

Structural probing performed *in vitro* on vRNA associated to tRNA_3_^Lys^ by thermal annealing [48] led to the same results as *in situ* probing on vRNA/tRNA complexes isolated from virions [45,48]. In contrast to the subtype A situation, only the interaction involving the PBS could be observed for subtype B, with no structural rearrangement accompanying tRNA_3_^Lys^ annealing [48]. Recently, two studies using the SHAPE technology were performed on genomic vRNA extracted from virions and deproteinized. Although they generally gave very similar results, the secondary structure models derived from these experiments vary in the PBS region [46,47]. Data from the first publication lead to the proposal of two additional primer/template interactions, on top of the PBS interaction: one between the A-rich sequence and the U-rich anticodon loop of tRNA_3_^Lys^, the other between the 3′ anticodon stem and part of the variable loop and a sequence upstream of the PBS, as suggested by Iwatani and co-workers [74]. The second set of data does not account for any primer/template interactions beside the one involving the PBS. The difference between the two sets of data may lie in a slightly different protocol for virus preparation and deproteinization.

It was suggested that in MAL the extended interactions with tRNA_3_^Lys^, which incidentally were also confirmed by *in situ* probing [48], are required to trigger the structural rearrangements generating the three-dimensional elements ultimately recognized by RT. These rearrangements would be necessary to prevent steric clashes. The absence of structural rearrangements in NL4-3/HxB2 was accounted for by the fact that essential structural elements such as helices 1 and 2 and the three single-stranded nucleotides at the junction between the PBS helix and helix 2, found in the MAL RNA/tRNA_3_^Lys^complex, preexist in subtype B vRNA prior to tRNA_3_^Lys^ annealing [44,48] (Figure 4c, d). In line with this interpretation, an 18-mer RNA oligonucleotide complementary to the PBS efficiently primed reverse transcription of NL4-3/HxB2 RNAs [75,6], but that of MAL RNA [53].

The possible role of the A-rich loop was nevertheless investigated in the two very similar HxB2 and NL4-3 RNAs, leading to unexpectedly divergent observations. The deletion of the AAAA sequence severely reduced initiation of reverse transcription [75,76]. However, the final amount of (-) ssDNA synthesis was found to be only modestly decreased, presumably due to the elimination of strong pausing sites after the addition of 11 to 14 nucleotides [76]. This deletion was also reported to have only a modest effect on (-) ssDNA synthesis in the presence of NCp [77]. By contrast, an AAAA to UUUU substitution on NL4-3 RNA was found to enhance the efficiency of (-) ssDNA synthesis, in the absence and presence of NCp [74]. It is possible that the deletion of the AAAA sequence induces structural perturbation of primer/template complex, thus explaining these differences.

#### 4.2.3. The PAS/anti-PAS interaction

An alternative intermolecular interaction between the TΨC loop in tRNA_3_^Lys^ and a conserved 8-nucleotide sequence within helix 1, downstream of the PBS (123GACUCUGG130) (Figure 4d, e), termed the Primer Activation Signal (PAS), was proposed to be important for regulated reverse transcription [43,50]. Mutation of the PAS sequence (mutant 2L, Figure 4d) strongly reduced the efficiency of the initiation of reverse transcription, while mutation of the opposite strand (mutant 2R, Figure 4d) enhanced reverse transcription, both *in vitro* and using virion-extracted primer/template complexes. The latter effect is most likely due to disruption of helix 1 and exposure of the PAS sequence [43,50]. The double mutation (2LR), theoretically restoring base-pairing, did not sustain efficient reverse transcription, suggesting that the sequence rather than the helical structure is important for reverse transcription. It was also shown that the efficiency of reverse transcription could be modulated by PAS mutations engineered to strengthen or weaken the interaction with the anti-PAS [78]. However these mutations not only interfere with the PAS/antiPAS interaction, but also affect the stability of helix 1 that is directly recognised by RT [44,48]. Recently, other sequences downstream of the PBS were reported to regulate initiation of reverse transcription *in vitro*, by affecting the accessibility of the PAS motif [79]. Finally, attempts to complement, *in vitro*, the 2L mutation in the PAS sequence by a corresponding change in the antiPAS of a synthetic tRNA_3_^Lys^ primer (2L-tRNA_3_^Lys^) were not successful [78]. The similarity between the PAS/anti-PAS interaction in HIV-1 and the interaction proposed for Rous sarcoma virus genomic RNA and its tRNA^Trp^ primer [80], together with the conservation of the PAS sequence among several retroviruses, led to the proposal that retroviral reverse transcription could be regulated by a common mechanism [78].

These conclusions were challenged by another study conducted on the same HxB2 vRNA variants [81]. In agreement with the previous studies [43,50], *in vitro* (-) ssDNA synthesis was severely affected by mutation 2L and was not restored by the double mutation 2LR, whether tRNA_3_^Lys^ or an 18-mer RNA oligoribonucleotide complementary to the PBS were used as primers. However, this reduction was correlated with enhanced pausing, while the initial rate of primer extension was unaffected [81]. On the contrary to previous results [43,50], mutation 2R was found to reduce (-) ssDNA synthesis, with a five-fold decrease of the initial rate of primer extension [79]. This observation is consistent with *in vitro* data from another group indicating that efficient tRNA_3_^Lys^-primed (-) ssDNA synthesis required the 24 nucleotides downstream of the PBS (which include the sequence concerned by mutation 2R) [74].

Chemical probing on wild-type and mutant vRNAs supported the existence of base-pairing forming helix 1 and its disruption by mutation 2L [50,81]. However, the effect of mutations on RNA structure could not be unequivocally interpreted, as there was no clear evidence that the wild type secondary structure was restored in mutant 2LR [81]. Structural rearrangements were also suggested in mutants 2L and 2R, keeping open the question of whether the reverse transcription defects might be attributed to incorrect folding or to the inability to form the PAS/antiPAS interaction [81].

#### 4.2.4. The interaction involving the tRNA anticodon stem and the variable loop

As mentioned above, efficient (-) ssDNA synthesis was shown to require the 24 nucleotides downstream of the PBS when using NL4-3 vRNA template and tRNA_3_^Lys^ as a primer [74]. The same template sequence was also found to be required with an all-RNA 18-mer primer (R18) complementary to the PBS or a chimeric primer containing nine deoxyribonucleotides at the 3′ end, but not with a DNA primer or a chimeric primer containing deoxyribonucleotides at the 5′ end. This was consistent with the fact that (-) ssDNA synthesis is sensitive to the nature of the helical conformation of the primer/template duplex [53–55,82]. Interestingly, the requirement for the downstream sequence is alleviated by the nucleocapsid protein (NCp) when natural or unmodified tRNA_3_^Lys^, but not the R18 primer, were used as primers [74]. Remarkably, mutation of nucleotides 142–148 complementary to the 3′ strand of the anticodon stem and the variable loop of tRNA_3_^Lys^ (Figure 4d, e) abolished the rescue by NCp in the absence of the downstream sequence [74]. Thus, NCp seems to be able to increase reverse transcription by facilitating extended interactions between tRNA_3_^Lys^ and upstream template sequences. These results address the question of the conditional requirement for highly conserved sequences forming helix 1, depending on the presence or absence of NCp. A possible explanation given by Iwatani *et al.* [74] is that helix 1 (Figure 4d) might be important for the maintenance of a structure stabilizing the initiation complex in the absence of NCp.

### 4.3. Biological significance of extended HIV-1 RNA / tRNA_3_^Lys^ interactions for virus replication

The importance of extended interactions for viral replication was addressed by two different approaches: 1) sequence deletions containing the elements proposed to participate to these interactions, and 2) investigation of HIV-1 variant viruses that use a non-self tRNA primer for reverse transcription.

#### 4.3.1. HIV-1 viruses carrying sequence deletions upstream and downstream of the PBS

The deletion of the A-rich loop (169AAAA172) in HxB2 RNA resulted in diminished levels of infectivity and reduced synthesis of viral DNA [75]. After long-term culture, 167GG168 were substituted by two As, restoring wild-type reverse transcription and replication levels [73]. These results argued in favour of the importance of the conserved A-rich loop in HIV-1 replication. Besides, an earlier study showed that a 26-nucleotide deletion in the 3′ part of the U5 region (Δ153–179) of NL4-3 vRNA produced a severe defect in infectivity. However, the deletion was found to affect integration but did not impair viral DNA synthesis in acutely infected cells, despite the absence of the A-rich sequence. This study also indicated that deletion of a 25-nucleotide fragment adjacent to the previous one (Δ127–152) had no detectable effect on virus replication [81]. Incidentally, this deletion contains seven nucleotides proposed to interact with the 3′ strand of the anticodon stem and variable loop of tRNA_3_^Lys^, and six of the eight nucleotides of the PAS sequence [83] (Figure 4).

#### 4.3.2. HIV-1 variant viruses that use a non-self tRNA primer for reverse transcription

Evidence for a role of the A-rich loop/anticodon interaction was brought by experiments showing that HIV-1HxB2 could replicate by stably utilizing non-cognate tRNAs as primers, provided that the PBS and the A-rich loop were simultaneously mutated to match both the 3′-end and the anticodon loop of the chosen tRNAs. This held true for tRNA^His^ [84–86], tRNA^Met^ [87,88], and tRNA_1,2_^Lys^ [89]. These studies, together with the A-loop deletion (see above), pointed to the biological importance of the A-rich sequence. However, the results might be biased by the presence of overlapping sequences important for other functions, like integration [83,90,91]. It was also proposed that mutations of the A-rich loop allowed stable usage of non-cognate tRNAs by disfavouring tRNA_3_^Lys^, rather than favouring alternate primers [92]. In addition, mutated viruses rapidly acquired additional mutations that improved their replication efficiencies [85–89,93], suggesting that the A-rich sequence/anticodon-loop interaction may not be sufficient for optimal initiation of reverse transcription. The replication defect of these viruses might also be linked to their inability to selectively package their non-self tRNA primers [94].

Nevertheless, a direct correlation between the evolution of mutants using tRNA^His^ in cell culture [85,86,93], their efficiency in initiating reverse transcription *in vitro,* and the structure of the primer/template complex was observed [95]. Indeed, viruses that acquired additional adaptive mutations allowing a more stable interaction with the tRNA primer replicated more rapidly and displayed nearly wild-type levels of reverse transcription *in vitro* [85,86]. In addition, probing experiments indicated that the mutated A-rich loop forms stable interactions with tRNA^His^ only in the presence of these adaptive mutations [95].

Attempts were also made to switch the tRNA primer usage by mutating the PAS sequence. In short term cultures, viruses containing the PBS-PAS double mutations introduced to enforce the use of non-self primers tRNA^Pro^ or tRNA_1,2_^Lys^ replicated more poorly than viruses with mutations in the PBS alone [96]. This observation is in line with *in vitro* experiments indicating that tRNA_1,2_^Lys^ primed reverse transcription of an HxB2 RNA template containing the same PBS-PAS double mutation with only ∼5 % efficiency compared to the wild-type situation [78]. However, when using primer/template complexes extracted from virions, the PBS-PAS double mutation restored tRNA_1,2_^Lys^ extension to wild type levels [79]. The origin of the difference in the effects of these mutations when using either *in vitro* annealed primer/template or primer/templates extracted from virions is unclear.

Multiple independent virus evolution experiments selected one PBS variant that was able to stably utilize tRNA_1,2_^Lys^ and restored high replication rates [96]. This mutant contained a second site mutation in the PAS sequence (U126C) that might stabilize the PAS/anti-PAS interaction by converting a G-U pair into a G-C pair [96]. Another second site mutation was identified within the RT gene (G3600A) that was responsible for a G490E change in the RNase H domain. Both mutations improved the replication capabilities of the mutated virus but only the U126C mutation was able to prevent reversion to the wild-type PBS [96].

Upon prolonged culture of a virus with the wild type PBS and a mutated PAS sequence, revertant viruses that partially overcame the reverse transcription defects were selected [43]. Interestingly, revertant viruses all acquired a single nucleotide substitution (C150U) that did not restore the PAS sequence but stimulated elongation of reverse transcription [43]. This observation fits with the proposal that mutations in the PAS affect elongation of reverse transcription rather than initiation [81].

The inability of HIV-1 to accommodate a non-self tRNA primer by substituting the PBS alone undoubtedly demonstrates the importance of additional interactions for the initiation of reverse transcription: MLV, for which no additional interactions have been described [97] easily utilises different tRNAs as primers [65], while avian sarcoma virus, for which an interaction between U5 and the TΨC loop has been proposed [80], does not [98]. Incidentally, HIV-1 has been shown to infrequently use tRNA_5a_^Lys^ as primer [99]; this tRNA only differs from tRNA_3_^Lys^ by one base-pair in the acceptor stem and has the potential to interacts with both the viral A-rich loop and the PAS sequence.

### 4.3.3. Reaching a consensus?

Although the large collection of findings reviewed above provide evidence that the complementarity between the PBS sequence and the 3′ part of the primer tRNA is not sufficient to determine primer usage, it is difficult to unambiguously rationalize the existence of precise additional interactions. The origin of apparent discrepancies is probably multiple and reflects the high complexity of the process. The most obvious explanation arises from (i) the existence of biases such as the nature of the strains used, (ii) the experimental parameters and tools utilised and (iii) unexpected effects of mutations. In particular, RNA mutations may interfere with a variety of steps other than the targeted one during the replication cycle. In addition the source of the interference can be direct (*i.e.*, directly altering a binding site) or indirect (*i.e.*, by triggering an undesired conformation that becomes inactive or induces a steric conflict). Even though a lot of publications already addressed the question of the structure of the complex mediating initiation of reverse transcription, several additional studies might be envisaged. For instance, whereas many detailed kinetic studies have been performed *in vitro* on the HIV-1 MAL isolate, *in vivo* studies of mutations affecting the proposed structure are yet to be performed. Similarly, the effects of mutations affecting the proposed PAS/anti-PAS interaction on the kinetics of reverse transcription have never been studied in infected cells.

The proposed mutually exclusive interactions might proceed transiently and be required at different steps of the replication cycle. Nevertheless, the results collected from experiments designed to probe the structure of subtype A and subtype B vRNA/tRNA complexes undoubtedly reveal an unexpected structural versatility of the HIV-1 initiation complex. The extraordinary plasticity of the vRNA and its capacity to adapt to a variety of constraints, in particular those imposed by mutations, offers the virus several solutions to precisely match RT and the viral RNA/tRNA primer complex in order to efficiently promote reverse transcription. As an example, the absence of interaction between the A-rich loop of NL4-3 and HxB2 vRNAs and the anticodon loop of tRNA_3_^Lys^ is at variance with both *in vitro* and *ex vivo* data showing that a double mutation of the PBS and A-rich loop allowed the use of tRNA^His^ as a primer for reverse transcription. It was proposed that in this case the loop-loop interaction was used to compensate a negative contribution of tRNA^His^ [95]. This would imply that all tRNAs are not equally suited to prime reverse transcription, in agreement with a study comparing non cognate tRNAs [100].

### 4.4. The case of HIV-2

**Figure 5.**
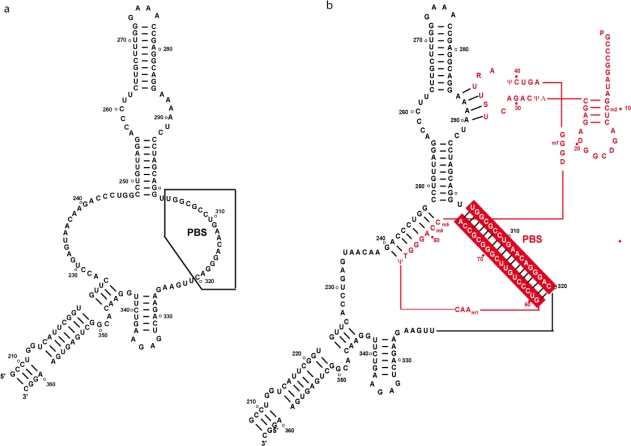
Secondary structure models of the HIV-2 vRNA (**a**) and of the vRNA/tRNA_3_^Lys^ complex (**b**). The tRNA is in red and the vRNA in black.

In the case of HIV-2, *in vitro* structural studies led to the proposal of a secondary structure in which most of the nucleotides of the vRNA are base-paired, with the noticeable exception of the PBS sequence, in which only 3 out of 18 nucleotides are involved in base-pairing interactions [101]. In this model, the PBS sequence is preceded by a long hairpin, containing two A-stretches in both an apical and an internal loop [101,102] (Figure 5a).

On the contrary to the situation with HIV-1 MAL, formation of the binary complex does not involve any intermolecular rearrangements [102] (Figure 5). However, similarly to HIV-1 MAL, the U-rich anticodon loop of tRNA_3_^Lys^ interacts with an A-rich loop upstream of the PBS, while nucleotides of the 5′ part of the TΨC stem interact with nucleotides in the U5 sequence upstream of the PBS [102], in a way reminiscent of the HIV-1 PAS/anti-PAS interaction (Figure 5).

## 5. Mechanism of primer tRNA annealing to HIV-1 RNA by the viral nucleocapsid protein

As described above, tRNAs are highly structured macromolecules. Formation of the binary vRNA/tRNA complex necessitates, at least, the unfolding of the 3D structure of the primer tRNA and melting of base-pairings in the acceptor and TΨC arms. Such a phenomenon does not occur spontaneously at 37 °C and numerous studies have identified the viral nucleocapsid protein as the required chaperone co-factor [77,103–106] (for a review see [107]). Annealing between the two partners of the reverse transcription initiation complex can be achieved either by the NC domain within the unprocessed pr55Gag precursor [108–112] or by the mature NC [77,103–106]. Mature NC is a small basic protein that exists mainly in two forms, NCp15 and NCp7, the latter being the final maturation product. It contains two CCHC zinc-fingers, separated by a short basic linker and flanked by N- and C-terminus basic residues [107]. The role of each NC domain in tRNA annealing has been the source of some debate but a consensus seems to have been finally reached. It is now accepted that although the zinc fingers are required for optimal chaperone activity [113–115], the basic amino-acids surrounding the N-terminal zinc fingers are absolutely necessary for the annealing activity [104,113,116]. Several groups have been trying to dissect the annealing process, using different approaches: terbium cleavages and kinetic studies for the Musier-Forsyth group and detection of base-pair melting or formation by NMR, in the absence or presence of NC, for the Dardel group. From their data, it appears that the first step of hybrid formation is the melting of a small duplex, followed by a nucleation step that brings together the sequences to be annealed [114,117]. The basic amino-acids flanking the first zinc finger, which do not directly contact the tRNA but strengthen binding, were proven to be essential for this process. Even though NMR studies showed that nucleation can occur without NCp, this protein dramatically accelerates the process and is strictly required for full annealing. The nucleation process could be initiated by the unpaired 3′ CCA of the tRNA [117] and/or by the four unpaired bases that form the junction between the acceptor and the TΨC-stems [114,118]. Unwinding of the rest of the double-stranded RNA regions happens thanks to the zinc fingers of NC, the structure of which is necessary for most of the specific contacts with tRNA [119]. The zinc fingers are also involved in destabilization of the tertiary TΨC/D loop interaction [113,120].

## 6. Effects of the restriction factors APOBEC-3G/3F and of the viral infectivity factor (Vif) on the early steps of reverse transcription

APOBEC-3G (A3G; APOlipoprotein B m-RNA-Editing enzyme, catalytic polypeptide 3G) and A3F are restriction factors expressed in primary human T-cells, macrophages and monocytes, which are the main reservoir of HIV-1 in humans, as well as in some lymphocyte-derived cell lines. The viral infectivity factor (Vif) neutralizes these restriction factors and is required for HIV-1 replication in these so-called “non-permissive” cells (for a review, see [121]). A3F and A3G are cytidine deaminases [122] that introduce C to U transitions during (-) strand DNA synthesis, leading to G to A mutations in the (+) strand DNA which are deleterious to the virus. It was long believed that the antiviral activity associated with APOBEC proteins was entirely due to cytidine deamination [121,123,124]. However, the basis for a possibly more complex mechanism were already laid by early observations by Dettenhoffer and Yu [125] who highlighted a more than 50% decrease in the tRNA_3_^Lys^-primed reaction in *vif* (-) virions produced from non-permissive cells. Accordingly, the antiviral activity of A3G has been correlated to the capacity of this protein to inhibit the synthesis of short reverse transcripts, rather than to hypermutation [126]. Indeed, a number of studies showed that enzymaticaly inactive A3G and A3F are able to inhibit HIV-1 reverse transcription, even though the deaminase activity is required for optimal anti-HIV-1 activity of these restriction factors [121].

A possible link with early steps of reverse transcription was suggested when an interplay between NC, A3G and RT was observed. Indeed, the NC domain of Gag is not only required for primer tRNA packaging and annealing, but also for A3G incorporation into the viral particles [127]. In addition, *in vitro* experiments indicated that A3G interacts with NC to inhibit tRNA annealing to the vRNA, and hence decreases priming efficiency by >50% *in vitro* and in cell culture [128,129]. The same inhibition features were described for A3F [130]. In protease (-) viruses, A3G does not inhibit the Gag-driven tRNA annealing to the PBS sequence. This hybridisation generates a vRNA/tRNA complex that is less stable than the complex formed later on, in the presence of mature NCp7 [110]. The initial “weak” vRNA/tRNA complex, formed by Gag, can be “rescued” *in vitro* into the mature complex by exposure to NCp7, and that rescue is inhibited by A3G [110]. The hypothesis developed by the authors is that the binary vRNA/tRNA complex formed in the presence of Gag, during the budding step, could be transformed by mature NCp7, within the mature virion, into a more solid complex that undergoes reverse transcription. In this context, the observation that Vif has an RNA chaperone activity and is able to promote annealing of tRNA_3_^Lys^ to the PBS *in vitro* may provide an additional mechanism by which Vif counteracts the antiviral activity of A3G and A3F [131]. However, inhibition of tRNA annealing by A3G was not confirmed by the other groups [132,133]. These studies suggested that A3G inhibits the elongation phase of reverse transcription, even in the absence of cytosine deaminase activity, but not tRNA_3_^Lys^ annealing, both *in vitro* [133] and when reverse transcription was performed in melittin-permabilized purified virions [132].

In conclusion, it has become clear recently that the reduced accumulation of viral DNA in HIV-1 infected cells in the absence of Vif is not only related to the cytidine deaminase activity of A3G and A3F but is likely linked to the inhibition of the early steps reverse transcription. Whether the annealing of tRNA_3_^Lys^ to the PBS and/or the initiation of reverse transcription are directly affected by these restriction factors awaits further experimental confirmation.

## 7. Spatio-temporal regulation of reverse transcription

### 7.1. In producer cells

Where and when exactly reverse transcription is initiated has been a debate for some time. Even though tRNA extended by two nucleotides has been isolated from purified virions [134], it was commonly accepted that the entire reverse transcription process could not take place within virions due to an insufficient dNTP concentration for completion of the reaction [135,136]. However, it was recently shown that low levels of complete reverse transcription products of genomic and spliced HIV-1 RNA could be detected in viral particles[137].

**Figure 6.**
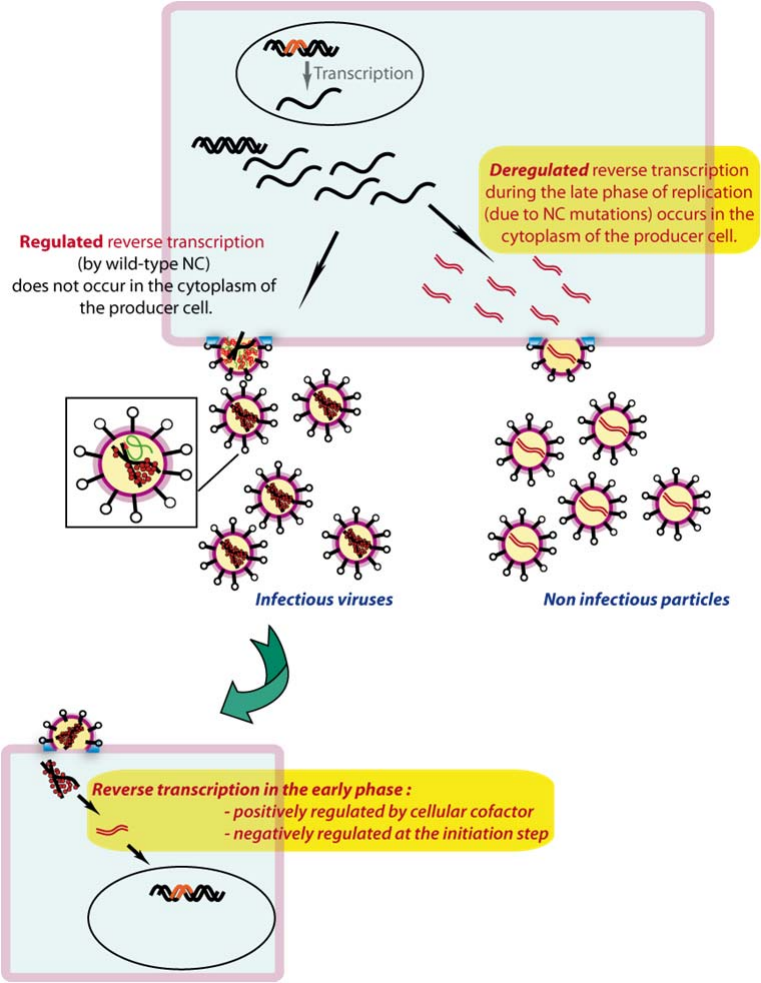
Schematic representation of the temporal regulation of reverse transcription in producer and target cells.

The chaperone NC protein not only plays a crucial role in annealing the primer tRNA to the vRNA, but is also involved in the control of the timing of reverse transcription during the late phase of the HIV-1 replication cycle. The latter property of NC was discovered recently, when Mougel and co-workers deleted one or both zinc fingers of NCp, leading to high levels of viral DNA packaged into viral particles that became non infectious [138] (Figure 6). It was demonstrated that DNA synthesis was not due to natural endogenous reverse transcription, but was indeed the first example of premature reverse transcription taking place during the late step of the replication cycle, in the producer cell. This result was confirmed by single mutations in one or the other zinc finger, as well as disruption of the NC/RNA interactions by mutation of the N-terminal basic residues of NCp, which all yielded late reverse transcription products [139,140]. Hence, wild type viral NC protein is required to ensure the correct timing of reverse transcription, since early reverse transcription during the late phase of the replication process, in the cytoplasm prior to budding, is detrimental to viral replication (Figure 6). Intramolecular base-pairing of the PAS sequence, which could modulate its interaction with tRNA_3_^Lys^, might play a similar role [43,50].

### 7.2. In target cells

Efficiency of reverse transcription is most likely also controlled during the early steps of the replication cycle, after viral entry. It was long believed that entry was immediately followed by an uncoating process that liberates the Reverse Transcription Complex (RTC) into the cytoplasm. Recent data from Charneau’s group suggested that the viral core remains assembled after entry and that reverse transcription takes place within that confined environment, keeping all the components tightly associated and moving along the microtubule network towards the nuclear compartment [141]. Uncoating, triggered by the synthesis of the central flap, would occur only after completion of reverse transcription and in close proximity to the nuclear pores [141,142]. The RTC would then transform into a pre-integration complex that is translocated through the nuclear membrane [141,142].

Recent work from the group of D. Harrich has shown that cellular factors stimulate reverse transcription in the target cells [143] (Figure 6). Unexpectedly, recent data from the same group showed that the early steps of reverse transcription are negatively regulated in the target cells and that suppression of this negative control is detrimental to HIV-1 replication (Figure 6) [144]. Thus, there is increasing evidence that initiation of reverse transcription is temporally regulated, both in the producer and in the target cells.

## 8. Initiation of reverse transcription as a drug target

All inhibitors that target the HIV-1 replication cycle have led to the development of resistance and this is true also for reverse transcription inhibitors. In the case of nucleoside analogues of reverse transcription (NRTI), resistance occurs either *via* increased discrimination of the NRTI against the natural dNTP or by phosphorolytic excision, the reverse reaction to polymerization, of the chain-terminating analogue [145–148]. Interestingly, it has been shown that AZT, the first and still one of the main NRTIs used in combination therapies, cannot be removed by phosphorolysis or ATP-lysis during the initiation phase of reverse transcription, by either wild type [149] or AZT-resistant RT [150]. The most likely explanation for this phenomenon is that the structure of the active site of RT containing the vRNA/tRNA complex is distorted and does not permit removal of the chain terminator in the same way as a for DNA/RNA or DNA/DNA hybrids. This makes the initiation complex a very interesting target for the development of specific inhibitors.

RNA, RNA/RNA and RNA/protein complexes can be targeted by small molecule (for review see [151]). The group of F. Dardel has pursued the idea of finding small molecules that could bind the primer tRNA_3_^Lys^ and hence destabilize the tRNA_3_^Lys^/vRNA initiation complex. An NMR screen, based on high selectivity, was used and three short peptides were selected, one of them interacting with tRNA_3_^Lys^ with a 2 mM dissociation constant. All three peptides were found to recognize the 3D structure of the D-stem and loop of tRNAs [152]. Compounds were further optimized for tRNA_3_^Lys^ binding [153,154]; their effect on initiation of reverse transcription needs now to be tested.

The strict requirement for the PBS sequence for all HIV-1 isolates and the importance of the A-rich loop for initiation of reverse transcription [44,53,66] led to the development of PNA antisense oligonucleotides [155,156] or 2′-O-methyl-antisense oligonucleotides [157] targeting the PBS and PNAs targeting the PBS together with the upstream A-rich loop [158] or the A-rich loop only [159]. All the above-cited antisense oligonucleotides strongly inhibited viral replication as well as initiation of reverse transcription *in vitro* and in endogenous reverse transcription assays in the case of PNAs. The potential of PNA molecules as candidates for the development of new drugs was also emphasized by the fact that their uptake is efficient in the presence of a membrane-transducing (MTD) peptide, with IC_50_ for inhibition of viral replication in the range of 0.5 to 0.75 μM [156]. Moreover, MTD peptide conjugates of PNAs also diplay virucidal activity [156].

RNA interference could also be a promising tool for antiviral therapy in general and anti-HIV therapy in particular [160–162]. The use of a combinatorial approach for shRNA therapy did yield some success in generating shRNA sequences that inhibit viral replication effectively [163]. However, although it was shown that viral escape was reduced when highly conserved sequence were targeted [164], targeting the PBS sequence using this strategy has not been successful [165].

## 9. Conclusions

Like most steps in HIV replication, initiation of reverse transcription has been the subject of a very large number of *in vitro* and *ex-vivo* studies.

The mechanisms allowing selective packaging of the tRNA_3_^Lys^ primer inside the viral particles are now well understood. Our understanding of the tRNA_3_^Lys^ annealing process mediated by the Gag precursor and the subsequent maturation of the initiation complex by mature NCp7 also significantly improved. New questions regarding tRNA_3_^Lys^ annealing emerged from the observations that A3G and A3F may inhibit this process. However, these findings have been questioned and await further confirmation. In the same context, the ability of Vif to replace NCp7 in the annealing reaction, and thus to potentially act as a counteracting factor to A3F and A3G, has been demonstrated *in vitro* but remains to be assessed *in vivo*.

Once the binary vRNA/tRNA_3_^Lys^ complex is formed, synthesis of the (-) strand strong stop DNA proceeds in two phases, initiation which corresponds to the addition of the first 6 nucleotides to the tRNA primer, and elongation, both of which have been well characterized from the enzymology point of view. On the contrary, the intermolecular interactions between the viral RNA and tRNA_3_^Lys^ taking place during this process have been a subject of debate and controversy. The structural versatility of the PBS region of the HIV genomic RNA, which has been shown to adopt different structures in different isolates, might explain some of the discrepancies. Moreover, some differences between experiments published by different laboratories could also arise from the fact that certain RNA conformations, even though important, are transient and/or that mutant RNAs may adopt a alternative structure not reflecting the wild-type situation.

Finally, one of the recent and fascinating advances in the field concerns regulation of reverse transcription, in space and in time. Indeed, it seems clear that tight negative regulation of reverse transcription in producer cells is crucial for the infectivity of the virus, but also that a controlled initiation of reverse transcription process in infected cells is important for proper replication.
